# Immunohistochemical Characterization of Three Monoclonal Antibodies Raised against the Epidermal Growth Factor and Its Receptor in Non-Small-Cell Lung Cancer: Their Potential Use in the Selection of Patients for Immunotherapy

**DOI:** 10.1155/2013/627845

**Published:** 2012-12-11

**Authors:** Charles E. Rengifo, Rancés Blanco, Damián Blanco, Mercedes Cedeño, Milagros Frómeta, Enrique Rengifo Calzado

**Affiliations:** ^1^Department of Pathology, Manuel Fajardo General Hospital, 10400 Havana, Cuba; ^2^Laboratory of Recognition and Biological Activity Assays, Department of Quality Control, Center of Molecular Immunology, Atabey, Playa, P.O. Box 16040, 11600 Havana, Cuba; ^3^Department of Cell Biology and Tissues Banking, National Institute of Oncology and Radiobiology, 10400 Havana, Cuba

## Abstract

Adequate methods to identify which lung cancer patients are most likely to benefit from the targeted drugs against both epidermal growth factor receptor/epidermal growth factor (EGFR/EGF) are needed. For this reason, we evaluated both the tissue reactivity of ior egf/r3 monoclonal antibody (Mab) in human lung carcinomas and its biological activity in NCI-H125 cells. Additionally, we assessed the tissue expression of EGF using two Mabs, CB-EGF1 and CB-EGF2. The overexpression of EGFR was detected in 33.33% and 62.71% of small-cell lung carcinoma (SCLC) and non-small-cell lung carcinoma (NSCLC), respectively. The ability of ior egf/r3 Mab to bind the extracellular domain of EGFR inhibiting cell proliferation and inducing apoptosis in NCI-H125 cells was also demonstrated. The EGF expression was observed in about 17% and 70% of SCLC and NSCLC, respectively. However, differences in the reactivity of CB-EGF1 and CB-EGF2 were evidenced. A dual expression of EGFR and EGF was observed in 16.67% and 57.63% of SCLC and NSCLC patients, respectively. But, a correlation between them was only obtained in NSCLC. Our results permit to recommend the development of diagnostic kits using ior egf/r3 and/or CB-EGF1 Mabs in order to achieve a better selection of patients to EGFR/EGF-targeting treatment.

## 1. Introduction

Lung cancer is one of the leading causes of cancer-related deaths worldwide [1]. Non-small-cell lung cancer (NSCLC) is the most common form of the disease, accounting for approximately 85% of all cases [2]. In patients with NSCLC, some genetic and regulatory abnormalities have been considered responsible for the tumor survival advantage [3], limiting the survival benefit provided by the standard therapeutic options available. Therefore, drugs targeting abnormal pathways could lead to more effective treatments for this often difficult disease [4].

Since the identification of some alterations in the expression of both epidermal growth factor/epidermal growth factor receptor (EGF/EGFR) in lung cancer pathogenesis, several therapeutic targeting agents have been employed for the treatment of lung tumors overexpressing these molecules [5, 6].

At least three different types of agent for EGF/EGFR inhibition are currently in use: tyrosine kinase inhibitors (TKIs), monoclonal antibodies (Mabs), and molecular cancer vaccines. Among them, nimotuzumab, a humanized therapeutic monoclonal antibody that neutralize the EGFR, and CIMAVax-EGF, a molecular vaccine that induces anti-EGF antibodies neutralizing endogenous EGF, have demonstrated promising results in patients with NSCLC, alone or combined with established modalities [5–7].

Nevertheless, methods to identify which patients are most likely to benefit from these targeted drugs are needed. In this way, the application of adequate immunohistochemical methods could permit a better evaluation of these molecules leading patients to a more appropriate therapeutic strategy. To date, one of the most explored pretreatment biomarkers is the status of the target molecules. 

For these reasons, in this work we evaluated the tissue reactivity of ior egf/r3 Mab, the murine counterpart of nimotuzumab [8], in lung carcinomas as well as, the ability of this Mab to bind the extracellular domain of EGFR inhibiting cell proliferation and inducing apoptosis in a NSCLC cell line. Additionally, we assessed the tissue expression of EGF ligand using two different Mabs: CB-EGF1 and CB-EGF2 [9].

## 2. Materials and Methods

### 2.1. Tissue Specimens and Previous Processing

A number of 71 routinely processed, formalin-fixed, and paraffin-embedded archival samples with diagnosis of lung cancer were obtained from the pathology department of the National Institute of Oncology and Radiobiology. The samples were taken after obtaining the approval consent by the institutional ethical committee. Five microns serial sections from each block were obtained in a Lizt 1512 micrometer and mounted on plus slides (Dako S2024, Carpinteria, USA). All sections were attached to the slide by heating in a 60°C oven for 1 h. Afterward the slides were kept at room temperature and they were used within 30 days.

The slides were dewaxed in xylene and rehydrated in decreasing ethanol series as usually and endogenous peroxidase activity was blocked with 0.03% hydrogen peroxide in methanol for 30 minutes at room temperature. Afterward all sections were washed in distilled water for 10 minutes and then were rinsed with TBS (Tris/saline buffer solution) for 5 minutes. 

### 2.2. Immunohistochemical Staining

In order to verify the quality of the formalin-fixed and paraffin-embedded tissues a monoclonal antibody that detects an epitope common to many cytokeratin (clone MN-116, Dako M0821, Carpinteria, USA) was used. The slides were pretreated with 10 mM sodium citrate buffer at pH 6.0 for 10 minutes in a microwave oven at 600 W. The rest of sections were placed in a humid chamber and pretreated with 0.4% pepsin in 0.1N hydrochloric acid solution at 37°C for 30 minutes. After pretreatments, the slides were washed gently in tap water and then with distilled water and TBS, as were described above. 

The samples were incubated with the primary mouse ior egf/r3 (anti-EGFR), CB-EGF1, CB-EGF2 (anti-EGF), and MN-116 Mabs for 1 h at room temperature. Negative controls were performed by substituting primary antibody for TBS and sections of colonic adenocarcinoma of known positively for these antigens were taken as positive control. After two rinses in TBS the slides were incubated with a rabbit anti-mouse biotinylated secondary antibody (Dako E0354, Carpinteria, USA) and ABComplex/HRP (Dako E0355, Carpinteria, USA) both for 30 minutes at room temperature dilution 1 : 100. Between incubations, slides were washed with TBS for 10 minutes. Afterward, enzymatic activity was visualized with DAB substrate chromogenic solution (Dako K3465, Carpinteria, USA) and the tissues were counterstained with Mayer's Hematoxylin (Dako S2020, Carpinteria, USA). The samples were dehydrated and mounted with a synthetic medium.

### 2.3. Immunohistochemical Evaluation

The IHC score (H-score) was used as was previously described [10]. Briefly, all markers were evaluated for percentage of positive cells (0–100%) and the intensity of reaction (0–3+). The results in agreement with two observers (ChER and RB) were considered as final. Afterward, the H-score was calculated for each specimen by multiplication of the intensity of reaction and the grade of positive cells, resulting in a score ranging from 0 to 300. Subsequently, these scores were grouped as follow: 0 (score 0); 1 (scores < 150); 2 (scores ≥150).

### 2.4. Pathological Features Evaluation

Some morphological parameters such as histopathological classification, grade of differentiation, degree of cellular pleomorphism, and mitotic and necrosis indexes were evaluated for an expert pathologist (ChER) in each tumor tissue using hematoxylin and eosin (H/E) staining.

The degree of cell pleomorphism was evaluated considering some cytomorphologic characteristics such as cell and nuclear size, cellular shape, chromatin pattern, nucleoli, and amount of cytoplasm, and was scored as follows: 0 (no evident cell pleomorphism), 1 (low), 2 (moderate), and 3 (high) cell pleomorphism. Mitotic activity index (MAI) was recorded by the evaluation of 10 high-power fields with 400x magnification (10x ocular, 40x objective) in the most cellular area of the tumor (containing the subjectively highest number of mitoses) [11]. Only unequivocal mitotic figures were counted. Finally, the MAI was calculated by dividing cells out of total cells counted and expressed as previously described for cell pleomorphism. For the evaluation of the degree of tumor necrosis (necrosis index) on each section a low-power field with 100x magnification (10x ocular, 10x objective) was used. It was scored subjectively as follows: 0 (no necrosis), 1 (less than 50% of necrosis areas per field), and 2 (more than 50% of necrosis areas per field). 

### 2.5. Cells Line and Culture

A human non-small-cell lung cancer NCI-H125 (ATCC CRL-5801) cell line was kindly provided by the research direction (Center of Molecular Immunology). Cells were grown in Dulbecco's modified Eagle's media (12800-017, GIBCO Invitrogen Co., USA) supplemented with 5% heat-inactivated fetal bovine serum (100082, GIBCO Invitrogen Co., USA). Cells were maintained at 37°C in a humidified atmosphere of air containing 5% CO_2_ and were harvested using a trypsin-EDTA solution (T-3924, SIGMA-ALDRICH Co. St. Louis, MO, USA).

### 2.6. Inhibition of Cell Proliferation and Apoptosis Measurement

Cells were seeded in a 6-well cell culture dishes at a density of 100.000 cells/well, in culture medium containing 5% FBS, and incubated at 37°C and 5% CO_2_. Twenty four hours later, the medium was changed to 1% FBS for serum starvation, and cells were exposed to different concentrations (12.5, 25, 50, 100, and 200 *μ*g/mL) of the murine anti-EGFR antibody ior egf/r3 for 96 h. An irrelevant Mab was used as negative control (T1 h Mab raised against CD6). Also, additional doses of each antibody were added when freshly medium was replaced according to the following experimental design: treatment 1 (no additional dose), treatment 2 (one additional dose after 48 h), and treatment 3 (additional doses every day). The cells were harvested by trypsinization, washed and resuspended in 0.5 mL FACSFlow (Becton-Dickinson, USA). Afterward, cells were incubated for 30 minutes with 0.5 mL of cold absolute ethanol (HS002-97003, Spectrum Chemical MFG Co., USA) at 4°C. For cell proliferation analysis, fixed cells were stained with propidium iodide/RNase solution for at least 30 minutes at room temperature. The percentage of cells in sub-G0-G1, G0-G1, S, and G2-M phases was analyzed using a FACScan flow cytometer (Becton-Dickinson, San Jose, CA, USA) equipped with a doublet discrimination module (DDM), CellQuest software (version 2.0) and ModFit LT (Verity Software House, version 2.0), respectively. To determine the apoptotic population, the sub-G0-G1 peak was also measured. Each experiment was repeated at least twice on different days using different sub-cultures of NCI-H125 cell line.

### 2.7. Statistical Analysis

GraphPad Prism 5 software (2007 GraphPad Software Inc. La Jolla, CA, USA) was used for data analysis. The correlation between the reactivity of CB-EGF1 and CB-EGF2 Mabs was assessed by Spearman ranks correlation coefficients. Survival distribution was estimated by the Kaplan-Meier method. Survival comparison was performed by two-sided log-rank tests. The percent of cells in sub-G0-G1, G0-G1, S, and G2-M phases of the cell cycle according to the experimental design was compared using Two-way ANOVA followed by Bonferroni post hoc test. For all tests, *P* < 0.05 was considered statistically significant. The percent of cell growth inhibition was calculated using the average of cell in S phase, according to the following formula: % of cell growth inhibition = (control sample − treatment sample)/control sample ×100. 

## 3. Results

### 3.1. Patient Description and Pathological Features

**Figure 1:**
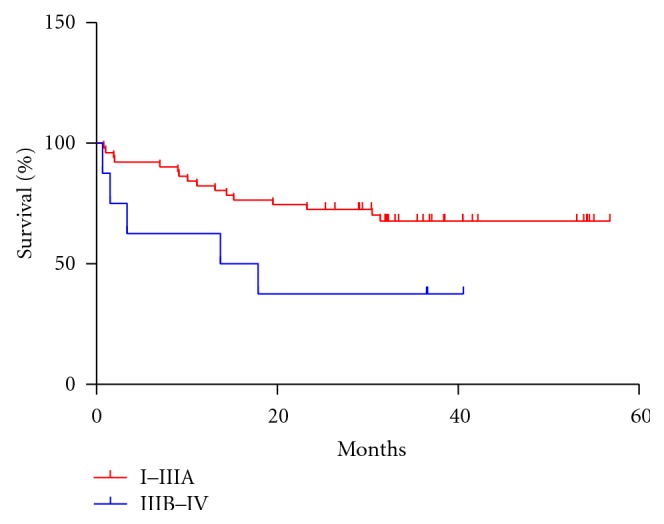
Kaplan-Meier estimate of overall survival among NSCLC patients showing different stages of the disease (*P* = 0.0405; Log rank test).

Tables 1 and 2 showed a summary of patient characteristics and some pathological features. The median patient age at presentation was 55.65 years (ranged from 23 to 86 years). Median overall survival of the population was 25.52 months (ranged from 0.93 to 57.17) for SCLC, 30.16 months (ranged from 0.70 to 40.57) for patients with stage I, II, or IIIA NSCLC, and 18.86 months for patients with stage IIIB or IV NSCLC. The stage was found to be associated with the overall survival of NSCLC patients (*P* = 0.0405; Log rank test) (Figure 1).

### 3.2. EGF Receptor Was Mainly Expressed in NSCLC but No Correlation with Survival Was Observed

**Figure 2:**
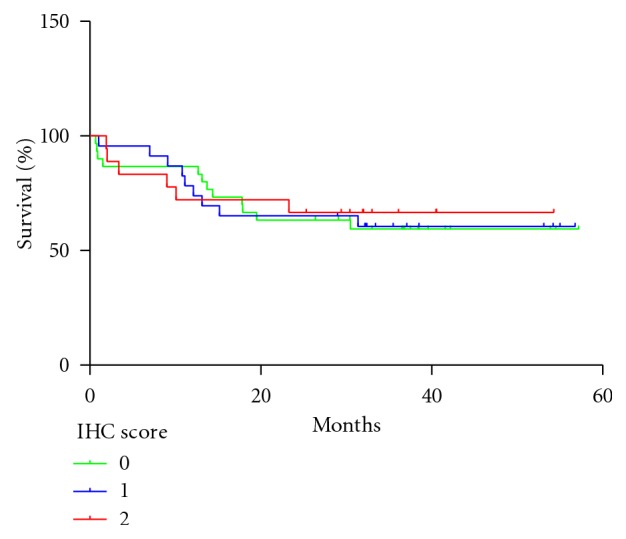
Kaplan-Meier estimate of overall survival among NSCLC patients showing different level of EGFR expression (*P* = 0.9560; Log rank test).

**Figure 3:**
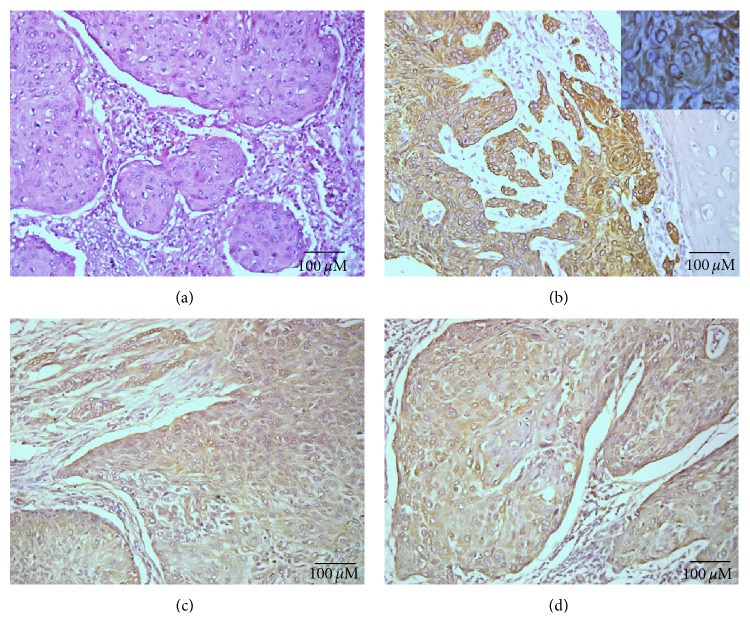
Hematoxylin and eosin staining of squamous cell carcinoma of the lung (a). Note: the intense reaction of ior egf/r3 Mab is mainly located in cell membrane and also in the cytoplasm of malignant epithelial cells (b) (inset on the upper-right corner, 1000x magnification). An intense immunostaining with both CB-EGF1 and CB-EGF2 Mabs was also evidenced (c and d, resp.). Black bar =100 *μ*m.

The tissues showed a good morphology, preserving undamaged the molecular antigenic determinants by means of the immunoreactivity of the monoclonal antibody against cytokeratin. No correlation between the EGFR expression and the overall survival of NSCLC patients was observed (*P* = 0.9560; Log rank test) (Figure 2) neither with tumor and patient characteristics (data not shown). Nevertheless, a statistically significant difference was obtained when the overexpression of EGFR in stage I–IIIA and IIIB–IV was compared (*P* = 0.0264; Chi-square test). The pattern of staining of ior egf/r3 Mab was finely granular and was mainly located in the plasmatic membrane of malignant cells, although their cytoplasm was also decorated (Figures 3(a) and 3(b)). The reactivity of ior egf/r3 Mab was evidenced in 37/59 (62.71%) of NSCLC samples. According to the histopathological classification, 11/20 (55.00%) squamous cell carcinoma, 13/23 (56.52%) adenocarcinoma, 5/6 (83.33%) large cell carcinoma, and 8/10 (80.00%) of other minor types represented were recognized by the ior egf/r3 (Table 3). The expression of EGFR was also evidenced in 4/12 (33.33%) of SCLC.

### 3.3. Expression of EGF Was Also Detected in Lung Tumors although Differences in the Reactivity of the Anti-EGF Mabs Were Evidenced

We used two different anti-EGF ligand monoclonal antibodies (kindly provided by the Center for Genetic Engineering and Biotechnology, Havana, Cuba). The percent of positive cases according to the histopathological classification of the tumors is extensely described in Table 4. The pattern of staining of these Mabs was finely granular and mainly located in cytoplasm, although a membrane staining was also observed. In addition, an extracellular pattern of staining was detected (Figures 3(c) and 3(d)). A slight increase in the intensity of reaction was observed with CB-EGF1 Mab. Noteworthy, a significant correlation was detected when the reactivity of CB-EGF1 and CB-EGF2 Mabs were compared (*P* < 0.0001, rs = 0.5429; Spearman test). However, no correlation between the EGF expression and the overall survival of patients was observed for both CB-EGF1 and CB-EGF2 Mabs (*P* = 0.9144 and *P* = 0.2706; Log rank test, resp.). No differences were also evidenced when the reactivity of both CB-EGF1 and CB-EGF2 Mabs were compared in stage I–IIIA and IIIB–IV (*P* = 0.8154 and *P* = 0.2743; Chi-square test, resp.).

### 3.4. The Dual Expression of Both EGFR and EGF Correlated in NSCLC but Not in SCLC

The dual expression of both EGFR and EGF was observed in 2/12 (16.67%) and 34/59 (57.63%) of SCLC and NSCLC, respectively. A significant correlation was detected when the expression of EGFR was compared with the reactivity of CB-EGF1 Mab in NSCLC samples but not in SCLC (*P* = 0.0006, rs = 0.4319 and *P* = 0.3784, rs = −0.2798, respectively; Spearman test). Similar results were obtained with CB-EGF2 Mab (*P* = 0.0035, rs = 0.3740 and *P* = 0.5918, rs = −0.1725; Spearman test). Additionally, no correlation between the dual expression of both EGFR and EGF with tumor and patient characteristics was obtained (data not shown) neither with the overall survival of NSCLC patients (*P* = 0.7321 and *P* = 0.9235; Log rank test).

### 3.5. Inhibition of NCI-H125 Cell Proliferation and Apoptosis Induction Was Time and Dose-Dependent

**Figure 4:**
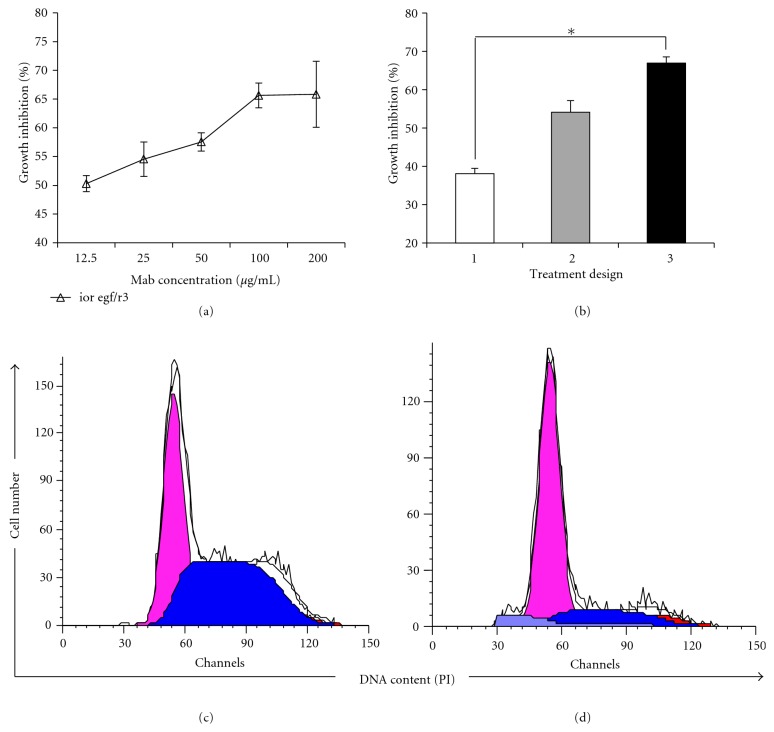
Antiproliferative activity of ior egf/r3 Mab in cultures of NCI-H125 cells. (a) Known numbers of NCI-H125 cells were treated with the indicated concentrations of anti-EGFR Mab and cultured for 72 h. The maximum of cell growth inhibition was obtained at 100 *μ*g/mL. Data points, means of triplicate samples; bars, SE. (b) Cells cultures were incubated for 72 with ior egf/r3 Mab at 100 *μ*g/mL following 3 different treatment designs (Materials and Methods). See: The statistical significant differences between treatment 1 and 3 (S phase, 0.0149; ANOVA). (c) and (d) Representative flow cytometry histograms of the NCI-H125 cells treated with the irrelevant Mab and with ior egf/r3 Mab, respectively. (d) Note an increase in the percentage of cells undergoing apoptosis (area in gray color) as well as the decrease in the percentage of cells in S phase (area in blue color).

We evaluated the ability of ior egf/r3 Mab to mediate specific biological functions such as cell growth inhibition and induction of apoptosis in a non-small-cell lung cancer cell line. Growth curve profiles were evaluated in NCI-H125 following the addition of different antibody concentrations of ior egf/r3 (12.5, 25, 50, 100 and 200 *μ*g/mL) in a period of 48, 72 and 96 h. No differences in the inhibition of cell proliferation were detected after 48 and 72 h (data not shown). The percent of inhibition ranged from 50.30 to 65.64% (S phase) as compared to the irrelevant control, obtaining the maximum of cell growth inhibition at 100 *μ*g/mL (Figure 4(a)). No induction of apoptosis was observed with this experimental design (sub G0-G1 peak, *P* = 0.3062; ANOVA).

To investigate whether the biological activity of ior egf/r3 is dose-dependent, additional doses of Mabs were added when freshly medium was replaced. This growth inhibition profile is showed in Figure 4(b). The addition of ior egf/r3 caused a significant inhibition on cell proliferation 48 h after Mab was added to the culture (treatment 2) (S phase, *P* = 0.0015; ANOVA), reaching the maximal inhibition at 96 h (treatment 3) (S phase, *P* = 0.0053; ANOVA), as compared to the treatment 1 (S phase, *P* = 0.0149; ANOVA) used as control. The exposure to ior egf/r3 Mab caused a more marked inhibition on proliferation in a dose-dependent manner. Curiously, after 96 h of incubation and adding additional doses of ior egf/r3 Mab (treatment 3), an increase in the percentage of cells undergoing apoptosis was obtained as compared to the irrelevant Mab (sub-G0-G1 peak, *P* = 0.0290; ANOVA) (Figures 4(c) and 4(d)).

## 4. Discussion

Up to date, TNM staging system (Tumor, Node, Metastasis) after surgery is considered the most important prognostic factor in NSCLC [12, 13]. TNM staging system also leads the clinicians to the selection of a more appropriate conventional treatment (e.g., surgery, radiation, chemotherapy) for NSCLC patients. In this study, we showed an increased overall survival of patients at stages I–IIIA as compared with the group of patients at stages IIIB–IV. It is known, the prognosis of patients at stages IIIB–IV NSCLC is poor due to the effectiveness of the conventional modalities of treatment. Therefore, the application of newer treatment modalities in order to improve patient survival and overall quality of life is mandatory [12, 13]. Consequently, in the last years the EGFR pathway inhibition has been accepted as an option for the first-, second- and third-line therapy of NSCLC [14]. 

In NSCLC, the overexpression of EGFR has been reported to be ranging from 40% to 89% [15, 16]. Here, we obtained the overexpression of EGFR in about 62% of NSCLC using the ior egf/r3 Mab. The ability of ior egf/r3 Mab to recognize human EGFR in frozen lung carcinoma tissues by immunohistochemistry was previously reported [17]. Moreover, the efficacy of the 99mTc-labeled ior egf/r3 Mab for the detection of epithelial-derived tumors, their metastases and recurrences by radioimmunoscintigraphy was previously evaluated [18]. In addition, no correlation between the expression of EGFR and the overall survival of patients was observed. The overexpression of EGFR has been associated with a more aggressive disease and reduced survival in a variety of tumors types [19], but in NSCLC the evidences are less convincing [20]. 

Additionally, we showed that blocking EGF binding to the receptor, in a NSCLC-derived cell line (NCI-H125) by means of ior egf/r3 significantly decreased tumor cell proliferation and induced apoptosis. The ior egf/r3 Mab recognizes an epitope located in the extracellular domain of the human EGFR with high affinity [21]. Nevertheless, it is known that therapy with murine-derived Mabs is limited by their tendency to develop human anti-mouse antibodies response (HAMA) [22]. For this reason, a humanized therapeutic version of ior egf/r3 Mab (nimotuzumab) was developed in our center [8]. In clinical trials, nimotuzumab has exhibited promising results used as a sensitizer to radio- or chemotherapy in advanced NSCLC patients positive for EGFR expression [23, 24]. 

Usually, the selection of patients to any anti-EGFR therapy using a neutralizing Mab is based on the tissue reactivity of a different anti-EGFR Mab for diagnostic purposes. On the contrary, nimotuzumab was obtained by transplanting the complementary determining regions (CDRs) of the ior egf/r3 Mab to a human framework [8]. Nimotuzumab and egf/r3 showed a very similar immunohistochemical pattern of recognition of fetal, adult and some malignant tissues, including SCLC and NSCLC [17]. In this way, the evaluation of EGFR expression by IHC using the ior egf/r3 Mab could provide more specific information in the selection of NSCLC patients to nimotuzumab treatment.

In a previous report, EGFR was significantly more expressed in stage IIIA when compared to earlier stages (I-II) [25], suggesting that expression increases stepwise from precancerous lesions to more advanced stages of cancer [26]. Nevertheless, in this work, patients at stage I–IIIA displayed higher level of EGFR expression as compared with those at stages IIIB–IV. Interestingly, we obtained a significant correlation when the expression of EGFR and EGF was compared, although, no differences in the expression of EGF between NSCLC stages were evidenced. It is known, the upregulation of EGFR expression by EGF is considered a mechanism that promotes the development and progression of tumors [27]. 

Our results permit to suggest a major activation state of the EGF/EGFR system in earlier stage of the disease, due to EGFR is mainly activated by the binding of its ligands. In line with this, some authors have suggested that growth factor/receptor loop is more important for lung tumor formation than for tumor progression [28, 29]. Notoriously, the dual expression of EGFR and EGF was observed in about 60% of NSCLC patients. In this way, our data also support the potential use of combined passive and active immunotherapy in tumors overexpressing both molecules, not depending of the stage of the disease.

On the other hand, the identification of deregulated expression of EGF family ligands in lung cancer pathogenesis has permitted to consider their potential use as therapeutic targeting agents [30]. Here, we found the tissue overexpression of EGF in at least the 70% of NSCLC samples, although no correlation between the EGF expression and the overall survival of patients was obtained. In a previous report, both increased levels of serum EGF as well as increased reactivity to EGF were found in NSCLC patients. Nevertheless, a correlation of higher level of serum EGF and poorly overall survival of patients was evidenced [6].

Nevertheless, we obtained statistical significant differences when reactivity of CB-EGF1 and CB-EGF2 Mabs were compared. In a previous report using competition assays, it was demonstrated that CB-EGF1 and CB-EGF2 bind to different antigenic determinants of EGF. In addition, differences in the specificity of these Mabs have been reported. While CB-EGF1 is a highly specific Mab to human EGF, the CB-EGF2 Mab also reacts with the murine EGF [9]. These results could support the major reactivity of CB-EGF1 Mab evidenced in our study. Interestingly, an increasing in the overall survival of advanced NSCLC patients treated with CIMAVax-EGF has been reported [6, 7]. In this way, the evaluation of EGF expression on both serum and tumor section using the CB-EGF1 Mab could lead to a better selection of NSCLC patients to CIMAVax-EGF therapy.

Finally, the EGFR is commonly overexpressed in NSCLC, but it is rare in SCLC [15, 31, 32]. In our study, we obtained the overexpression of EGFR in about 33% of SCLC. By the contrary, the immunostaining was observed mainly located in the plasmatic membrane of malignant cells. However, Schmid et al. reported a cytoplasmatic and membranous staining of EGFR in SCLC [33]. Additionally, we detected the expression of EGF in about 17% of tumors, although no correlation between the expression of EGFR and EGF was detected. Previously, Kaseda et al. published both no EGF binding activity in 6 SCLC using 125I-EGF joint to no EGFR amplification in those specimens tissues [34]. Nevertheless, the activity of the EGFR pathway in SCLC has been previously demonstrated [33]. In spite of the fact that our data is very small; the exploration of the potential use of both EGFR and EGF as target for SCLC immunotherapy could be of interest. In this way, experiments in order to evaluate the functionability of the EGF/EGFR system in SCLC are being planned in our laboratory.

## 5. Conclusions

In summary, we reported the tissue reactivity of ior egf/r3 Mab, the murine counterpart of nimotuzumab, in both SCLC and NSCLC. The anti-proliferative activity and the capacity to induce apoptosis of egf/r3 Mab in a NSCLC cell line after binding the extracellular domain of EGFR were also demonstrated. In addition, we showed the immunohistochemical recognition of two different anti-EGF ligand monoclonal antibodies as well as their correlation with the expression of EGFR. The dual expression of both EGF and EGFR support the potential use of passive and active immunotherapy against these molecules alone or combined with established modalities. Moreover, our data permit to consider the development of diagnostic kits using ior egf/r3 Mab and CB-EGF1 Mabs in order to a better selection of patients to nimotuzumab and CIMAVax-EGF treatments, respectively.

## Figures and Tables

**Table 1:** Patients characteristic.

Features	No. (%)
Gender	
Female	23/71 (32.39)
Male	48/71 (67.61)
Age (years)	
<60	52/71 (73.24)
60–70	14/71 (19.72)
>70	5/71 (7.04)
Tumor size (cm)	
<3	27/71 (38.03)
>3	44/71 (61.97)
Tumor stage	
Small cell lung carcinoma	
LD	10/12 (83.33)
I	2/12 (16.66)
Non-small cell lung carcinoma	
LD	2/59 (3.39)
I	38/59 (64.41)
II	7/59 (11.86)
IIIA	4/59 (6.78)
IIIB	2/59 (3.39)
IV	6/59 (10.17)
Recurrence	
Yes	58/71 (81.69)
No	13/71 (18.31)
Overall survival	
Life	44/71 (61.97)
Death	27/71 (38.03)

No.: number of cases; %: percentages; LD: limited disease.

**Table 2:** Tumor characteristic.

Features	No. (%)
Histopathological type	
Small cell lung carcinoma	12/71 (16.90)
Non-small cell lung carcinoma	
Squamous cell carcinoma	20/71 (28.17)
Adenocarcinoma	23/71 (32.39)
Large cell carcinoma	6/71 (8.45)
Other	10/71 (14.08)
Grade of differentiation	
Well	10/71 (14.08)
Moderate	24/71 (33.80)
Poor	22/71 (30.99)
Undifferentiated	15/71 (21.13)
Degree of cell pleomorphism	
No evident	3/71 (4.22)
Low	29/71 (40.85)
Moderate	23/71 (32.39)
High	16/71 (22.53)
Necrosis index	
No evident	27/71 (38.03)
<50%	17/71 (23.94)
>50%	27/71 (38.03)
Mitotic index	
No evident	8/71 (11.27)
Low	22/71 (30.99)
Moderate	22/71 (30.99)
High	19/71 (26.76)

No.: number of cases; %: percentages.

**Table 3:** Immunohistochemical expression of EGF receptor.

		H-score	
Histopathological type	0	1	2
	No. (%)	No. (%)	No. (%)
SCLC	8/12 (66.67)	3/12 (25.00)	1/12 (8.33)
NSCLC			
Squamous cell carcinoma	9/20 (45.00)	6/20 (30.00)	5/20 (25.00)
Adenocarcinoma	10/23 (43.48)	7/23 (30.43)	6/23 (26.09)
Large cell carcinoma	1/6 (16.67)	3/6 (50.00)	2/6 (33.33)
Other	2/10 (20.00)	4/10 (40.00)	4/100 (40.00)

SCLC: small cell lung cancer; NSCLC: non-small cell lung cancer; No.: number of cases; %: percentages; 0: negative; 1: scores <150; 2: scores ≥150.

**Table 4:** Immunolocalization of EGF ligand using 2 different monoclonal antibodies.

	H-score		
Histopathological type	0	1	2
	No. (%)	No. (%)	No. (%)
SCLC			
CB-EGF1	2/12 (16.67)	6/12 (50.0)	4/12 (33.33)
CB-EGF2	5/12 (41.67)	5/12 (41.67)	2/12 (16.67)
NSCLC			
Squamous cell carcinoma			
CB-EGF1	3/20 (15.00)	12/20 (60.00)	5/20 (25.00)
CB-EGF2	6/20 (30.00)	13/20 (65.00)	1/20 (5.00)
Adenocarcinoma			
CB-EGF1	5/23 (21.74)	9/23 (39.13)	9/23 (39.13)
CB-EGF2	8/23 (34.78)	10/23 (43.48)	5/23 (21.74)
Large cell carcinoma			
CB-EGF1	1/6 (16.67)	2/6 (33.33)	3/6 (50.00)
CB-EGF2	0/6 (00.00)	3/6 (50.00)	3/6 (50.00)
Other			
CB-EGF1	3/10 (30.00)	1/10 (10.00)	6/10 (60.00)
CB-EGF2	4/10 (40.00)	3/10 (30.00)	3/10 (30.00)

SCLC: small cell lung cancer; NSCLC: non-small cell lung cancer; No.: number of cases; %: percentages; 0: negative; 1: scores <150; 2: scores ≥150.
